# Detection of a reduced susceptibility to chlorfenapyr in the malaria vector *Anopheles gambiae* contrasts with full susceptibility in *Anopheles funestus* across Africa

**DOI:** 10.1038/s41598-023-29605-w

**Published:** 2023-02-09

**Authors:** Magellan Tchouakui, Tatiane Assatse, Hervé R. Tazokong, Ambrose Oruni, Benjamin D. Menze, Daniel Nguiffo-Nguete, Leon M. J. Mugenzi, Jonathan Kayondo, Francis Watsenga, Themba Mzilahowa, Michael Osae, Charles S. Wondji

**Affiliations:** 1 Medical Entomology Department, Centre for Research in Infectious Diseases (CRID), P.O. Box 13501, Yaoundé, Cameroon; 2 Parasitology and Ecology Laboratory, Department of Animal Biology and Physiology, Faculty of Science, University of Yaoundé 1, P.O. Box 812, Yaoundé, Cameroon; 3 Entomology Department, Uganda Virus Research Institute (UVRI), P.O.Box 49, Entebbe, Uganda; 4 Institut National de Recherche Biomédicale, P.O Box 1197, Kinshasa, Democratic Republic of Congo; 5 Entomology Department, Malaria Alert Centre (MAC), Kamuzu University of Health Sciences (KUHeS), P.O Box 265, Blantyre, Malawi; 6 Radiation Entomology and Pest Management Centre, Ghana Atomic Energy Commission, Legon, PO Box LG80, Accra, Ghana; 7 Department of Vector Biology, Liverpool School of Tropical Medicine, Pembroke Place, Liverpool, L35QA UK; 8 International Institute of Tropical Agriculture (IITA), P.O. Box 2008, Yaoundé, Cameroon

**Keywords:** Infectious diseases, Genetics, Molecular biology

## Abstract

New insecticides have recently been produced to help control pyrethroid-resistant malaria vectors including the pyrrole, chlorfenapyr. Monitoring the susceptibility of mosquito populations against this new product and potential cross-resistance with current insecticides is vital for better resistance management. In this study, we assessed the resistance status of the major malaria vectors *Anopheles gambiae* and *Anopheles funestus* to chlorfenapyr across Africa and explored potential cross-resistance with known pyrethroid resistance markers. Efficacy of chlorfenapyr 100 µg/ml against *An. gambiae* and *An. funestus* from five Cameroonian locations, the Democratic Republic of Congo, Ghana, Uganda, and Malawi was assessed using CDC bottle assays. Synergist assays were performed with PBO (4%), DEM (8%) and DEF (0.25%) and several pyrethroid-resistant markers were genotyped in both species to assess potential cross-resistance between pyrethroids and chlorfenapyr. Resistance to chlorfenapyr was detected in *An. gambiae* populations from DRC (Kinshasa) (mortality rate: 64.3 ± 7.1%) Ghana (Obuasi) (65.9 ± 7.4%), Cameroon (Mangoum; 75.2 ± 7.7% and Nkolondom; 86.1 ± 7.4). In contrast, all *An. funestus* populations were fully susceptible. A negative association was observed between the L1014F-*kdr* mutation and chlorfenapyr resistance with a greater frequency of homozygote resistant mosquitoes among the dead mosquitoes after exposure compared to alive (OR 0.5; P = 0.02) whereas no association was found between GSTe2 (I114T in *An. gambiae*; L119F in *An. funestus*) and resistance to chlorfenapyr. A significant increase of mortality to chlorfenapyr 10 µg/ml was observed in *An. funestus* after to PBO, DEM and DEF whereas a trend for a decreased mortality was observed in *An. gambiae* after PBO pre-exposure. This study reveals a greater risk of chlorfenapyr resistance in *An. gambiae* populations than in *An. funestus.* However, the higher susceptibility in *kdr-resistant* mosquitoes points to higher efficacy of chlorfenapyr against the widespread kdr-based pyrethroid resistance.

## Introduction

Malaria is a major public health problem in sub-Saharan Africa. Although important gains have been achieved in reducing malaria burden since 2000^1^, pyrethroids resistance has emerged as a major obstacle to the global fight against the disease^2^. To mitigate the risk, recent novel non-pyrethroid insecticides or repurposed ones from agriculture have been developed to control malaria vectors^3,4^. These new molecules have unique toxicity mechanisms, relying mainly on mosquito physiology not on “usual” neurological or simple detoxification pathways. Among these products, there is chlorfenapyr, a new insecticide class (pyrrole) acting by disrupting respiratory pathways and proton gradients through the uncoupling of oxidative phosphorylation in mitochondria^5^ to exert mosquito mortality^6^. Chlorfenapyr inhibits the production of energy in mosquito’s mitochondria which consequently affects crucial and vital functions until eventual death^5^. This mode of action on an insect’s metabolism is particularly relevant for the control of vectors harboring metabolic insecticide resistance mechanisms such as cytochrome P450, glutathione S-transferases as increased metabolic activity increases the activation of the toxin and increase mosquito mortality^6^. This insecticide generally presents delayed toxicity when insects are inactive or constrained to a cage as these new physiological insecticides depend on the mosquito metabolism to act. This makes their evaluation very challenging with conventional neuro-toxic tests like WHO cone bioassay^7,8^. As this insecticide is bio activated by metabolic enzymes, evaluating the effect of metabolic-based pyrethroid resistance on the efficacy of chlorfenapyr could help improve vector control.

Compared to target-site resistance, metabolic detoxification is very common in mosquitoes and considered to be more likely to cause control failure^9^. The first phase of metabolic resistance involves the cytochrome P450 (CYP) enzyme system and other detoxifying metabolic enzymes that catalyse the oxidation reaction of insecticides; the second stage involves uridine diphosphate (UDP) glucosyltransferases (UGTs), which form various conjugated metabolites through conjugation; finally, in the third phase, the ATP-binding cassette transporters (ABC transporters) are involved in eliminating the second-stage metabolites from the cells^10^. In *An. funestus*, metabolic resistance to pyrethroids is mainly driven by CYP6s and CYP9s genes family such as *CYP6P9a, CYP6P9b, CYP6K1, CYP4M7, CYP325A, CYP9J11, CYP9K1, CYP6P5, CYP6P4a/b* and GSTs such as *GSTe2*^11–14^. In *An. gambiae*, this is mainly driven by *CYP6P3, CYP6P4, CYP6M2,* and *GSTe2*^15,16^*.* Only limited molecular diagnostic tools are currently available to detect metabolic resistance in field mosquitoes preventing to assess its impact on the efficacy of vector control tools. It was shown for example in *An. funestus* from southern Africa that the *CYP6AA1* had the ability to metabolise various types of pyrethroids^17^, while the overexpression of *CYP6Z1* is associated with cross-resistance against pyrethroids and carbamates^18^. In *An. gambiae*, overexpression of *CYP6P3* has been shown to be responsible for cross-resistance between pyrethroids and carbamates, and *CYP6M2* is linked to cross-resistance of pyrethroids and organophosphates^11,13,19^. Moreover, it was shown that the *CYP9K1* can metabolise deltamethrin and pyriproxyfen^20^. If such positive cross-resistance is seen with chlorfenapyr that will reduce the effectiveness of chlorfenapyr but if there is a negative cross–resistance, this will be very interesting as pyrethroid-resistant mosquitoes will be more vulnerable to the new active ingredient. Previous studies suggest that PBO has antagonistic effect on the toxicity of chlorfenapyr against *Culex quinquefasciatus* and *An. stephensi* mainly because it is bioactivated by cytochrome P450s and by blocking these enzymes, chlorfenapyr is not metabolised to its toxic form but just excreted. However, there is no clear evidence of negative association between P450-based or GSTs-based pyrethroid resistance and chlorfenapyr in mosquitoes probably due to the absence of DNA-based marker for metabolic resistance. This should be a critical step before the implementation of chlorfenapyr-based tool in the field. In this study, we comparatively assessed the susceptibility status of the major malaria vectors *An. gambiae* and *An. funestus* to chlorfenapyr across Africa and explored potential cross-resistance with known pyrethroid molecular markers.

## Methods

### Study sites

Mosquitoes were collected in five agricultural settings in Cameron (Mangoum, Nkolondom, Njombe-pendja, Mibellon and Elende) from May to July 2021, in one locality in 2021 from DR Congo (Ndjili Brasserie, a suburb of Kinshasa: 4° 19′ 39″ S, 15° 18′ 48″ E), Uganda (Mayuge: 0° 23′ 10.8′′ N, 33° 37′ 16.5′′ E); Ghana (Atatem: 5° 56′ N, 1° 37′ W) and Malawi (Chikwawa: (16° 1′ S; 34° 47′ E). In Cameroon, immature stages of *An. gambiae* were collected from the breeding site using the dipping method whereas adult *An. funestus* and *An.* gambiae from other countries were collected using electric aspirators. Two-five days old virgin females (F_1_ from field collected F_0_ or emerging F_0_ from collected larvae) were used for the bioassays.

### Molecular identification

Members of the *An. gambiae* complex were identified by the SINE-PCR^26,27^ after genomic DNA extraction with the Livak method^25^ whereas those from *An. funestus* group were analysed using cocktail PCR described by Koekemoer et al.^21^.

### Insecticide formulation

Technical-grade active ingredients was supplied by Sigma (PESTANAL^®^, analytical standard, Sigma-Aldrich, Dorset, United Kingdom). Stock solution was prepared by diluting the active ingredient in acetone or absolute ethanol, and storing in 50 ml falcon tubes, wrapped in aluminum foil, and at 4 °C. Working solutions were prepared using 1 ml of the stock solution. Field collected mosquitoes from the various sites were exposed to the insecticide in CDC bottle assay.

### Determination of susceptibility to chlorfenapyr

Approximately 24 h after coating bottles with insecticide, 25 female (2–5 days old) were exposed to chlorfenapyr 100 µg/ml for 1 h and the knocked-down mosquitoes were recorded at the end of the 60 min (Kd-60) exposure period. After recording the Kd-60 mosquitoes were gently aspirated from the bottle into clean paper cups and provided with 10% sugar solution soaked in cotton wool during the recovery period and the mortality was recorded 24 h, 48 h and 72 h post-exposure. In addition to the testing with the diagnostic dose of 100 µg/ml reported in literature^22^, ranges of insecticide concentrations (0, 10, 20, 30, 40, 50, and 100 µg/ml) were tested on the susceptible lab strain *Kisumu* and the LC_50_ obtained was used for comparison of susceptibility profile between different populations of *An. funestus*. Time exposure was performed also against field anopheles mosquitoes using the diagnostic dose of chlorfenapyr (100 µg/ml) in order to compare the LT_50_ between different populations.

### Evaluating the potential antagonistic/synergistic effect of piperonyl butoxide (PBO), di-ethyl maleate (DEM) and s,s,s-tri-butylphosphorotrithioate (DEF) on susceptibility to chlorfenapyr

To evaluate whether the inhibition of some enzyme systems could reduce susceptibility to chlorfenapyr, mosquitoes were pre-exposed to inhibitors of P450s (4% PBO), GSTs (8% DEM) or esterases (0.25% DEF) for 1 h, followed by 1 h exposure to chlorfenapyr (10 µg/ml for *An. funestus* and 100 µg/ml for *An gambiae*). Mortality was recorded 24 h, 48 h and 72 h after exposure and the differences in mortalities between inhibited and non-inhibited experiments were compared using a Chi-square test.

### Genotyping of pyrethroid markers for potential cross-resistance with chlorfenapyr

To assess the potential cross-resistance between chlorfenapyr and pyrethroids in *An. gambiae*, we crossed the highly resistant field strain from Nkolondom with the fully susceptible laboratory strain Kisumu. This hybrid (F_4_) strain were exposed to sublethal dose of chlorfenapyr to select the dead and alive mosquitoes. These mosquitoes were genotyped for the L1014F target-site knockdown resistance (Kdrw) and I114T-GSTe2 mutation all associated with DDT/pyrethroid resistance in *An. gambiae* using a Taqman (Santa Clara, CA, USA) method as previously described^23,24^. In *An. funestus*, the L119F-GSTe2 mutation was genotyped in mosquitoes from Elende using the AS-PCR method as described recently^25^.

Odds ratio and Fisher exact test were used to establish statistical significance of any association between this DDT/pyrethroid resistance markers and the ability of mosquitoes to survive chlorfenapyr exposure.

## Results

### Molecular identification of mosquitoes tested

PCR assays revealed that all the *An. gambiae* s.l tested from Mangoum (44/44), Nkolondom (50/50), and Congo (60/60) were *An. gambiae*. Those collected in Njombe were mainly *An. coluzzii* (60/60). The *An. gambiae* sl population from Uganda was a mix with 83% *An. gambiae* and 17% *An. arabiensis* whereas those from Ghana were 60% (39/65) *An. gambiae* and 40% (39/65) *An. coluzzii.* Molecular identification of a subset (n = 50) of F_0_ females *An. funestus* s.l. from Mibellon revealed that 45 (90%) were *An. funestus s.s.,* 3 (6%) were identified as hybrid *An. funestus/An. rivulorum-*like and 2 (4%) were *An. rivulorum*-like. Concerning the *An. funestus* collected from Elende*,* 100% (72/72) were *An. funestus s.s.* The Malawi *An. funestus* populations were at 91.1% *An. funestus* s.s and the remaining was other species of the group (4.54% *An. parensis*, 1.77% *An. rivulorum*) and hybrids (2.33%).

### Diagnostic dosage of chlorfenapyr using the susceptible lab strain Kisumu

**Figure 1:**
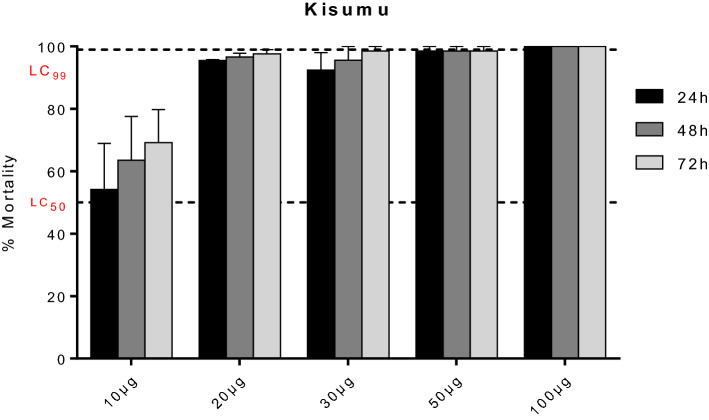
Assessment of diagnostic dose of chlorfenapyr using the susceptible lab strain Kisumu. Percentage mortality (24 h, 48 h and 72 h) of the susceptible lab strain Kisumu after exposure to chlorfenapyr (with acetone as solvent). LC50 represents the concentration able to kill 50% of mosquitoes and LC99 the concentration able to kill 99%.

Figure 1 summarises the mortality of Kisumu after exposure to chlorfenapyr. The LC_50_ and LC_99_ for both insecticides are represented on the same figure. No knock down was observed irrespective of the solvent used. The LC_50_ was approximately 10 µg/ml 24 h post-exposure whereas the LC_99_ within the 24 h was obtained with 50 µg/ml.

**Figure 2:**
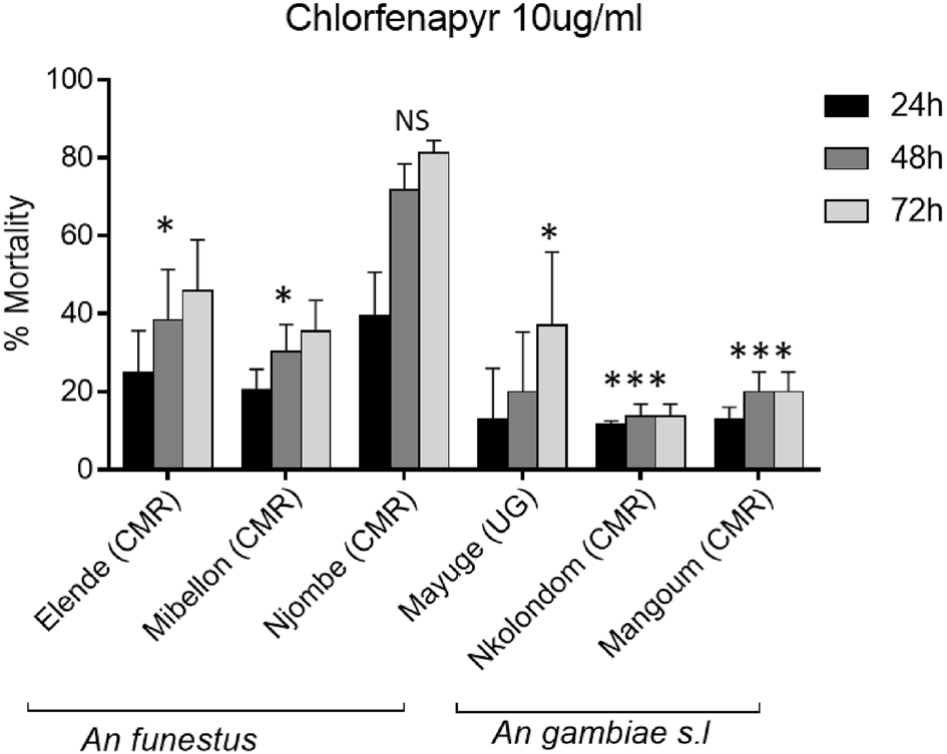
Comparative susceptibility pattern of field *An. gambiae* and *An. funestus* using the LC_50_ obtained with kisumu. Percentage mortality (24 h, 48 h and 72 h) after exposure to chlorfenapyr 10 µg/ml (with acetone as solvent); * indicate significant difference compared to Kisumu.

Exposure of the field collected samples to chlorfenapyr 10 µg/ml (the LC_50_ obtained with Kisumu) revealed lower mortality in all the field samples compared to Kisumu (Fig. 2) expect in Njombe-penja (81.3 ± 3.1%) where the difference was not significant (χ^2^ = 3.9; P > 0.05). Compared to Kisumu (69.2% ± 10.6%), the mortality rate after 72 h to chlorfenapyr 10 µg/ml was lower (13.3 ± 3.1) in *An. gambiae* from Nkolondom (χ^2^ = 64.4; P < 0.0001) and 20.0 ± 5.0 in *An. gambiae* from Mangoum (χ^2^ = 48.7; P < 0.0001). A slight increase in mortality rate was observed in *An. gambiae* from Mayuge (37.04 ± 18.7) and *An. funestus* from Mibellon (35.5 ± 7.9) and Elende (45.8 ± 13.4). All this showed that *An. gambiae* from Mangoum and Nkolondom which presented the lowest mortality (Fig. 2) are the less susceptible and that these populations are more likely to develop chlorfenapyr resistance.

### Susceptibility profile to chlorfenapyr 100 µg using acetone and absolute ethanol as solvents

**Figure 3:**
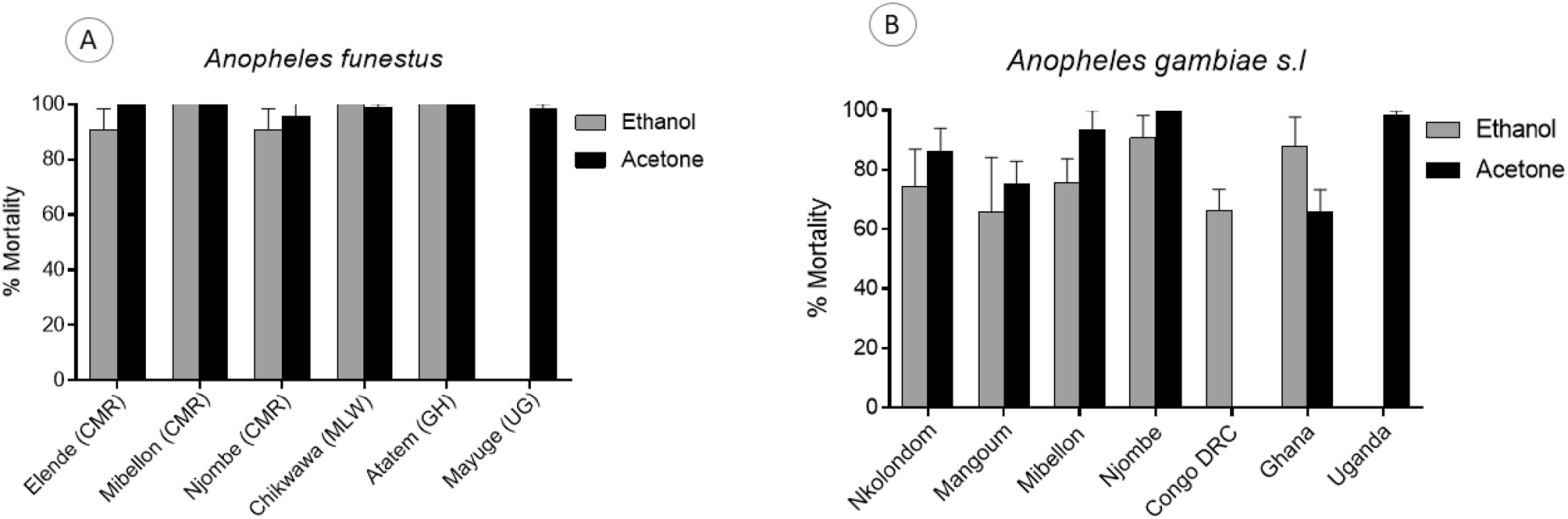
Susceptibility profile of *An. funestus* and *An. gambiae s.l* to chlorfenapyr 100 µg/ml across Africa. Mortality rate of *An. funestus* (**A**) and *An. gambiae* (**B**) from different sites 72 h after exposure to chlorfenapyr 100 µg/ml.

As the dose of 100 µg is the one recommended for routine evalutation of susceptibility to chlorfenapyr in malaria vectors in the field, we charaterised 10 populations including *An. gambiae* and *An. funestus* using both acetone and absolute ethanol. The mortality rate at this dose for the two solvents is summarised in the Table S1 showing a resistance trend in most of the *An. gambiae* populations and slight high efficacy of acetone compared to absolute ethanol. Most of the *An. funestus* populations were susceptible to this dose of chlorfenapyr when diluted in acetone but with ethanol some population (Njombe penja and Elende) showed a slightly reduced mortality rate, although not significant (Fig. 3A). In *An. gambiae*, varying levels of susceptibility was observed for the two solvents used (Fig. 3B; Table S1) with slightly reduced mortality rate observed with absolute ethanol as solvent compared to acetone. *An. gambiae* from Nkolondom, DR Congo and Mangoum had the lowest mortality rate with chlorfenapyr 100 µg/ml diluted in absolute ethanol with mortality rates of 58.2 ± 18.4%, 64.3 ± 7.1%, and 65.7 ± 18.5% respectively follow by *An. gambiae* from Mibellon with 75.6 ± 8.2% mortility (Fig. 3B). When using acetone as solvent, *An. gambiae* from Ghana had the lowest mortality (65.9 ± 7.4%) rate followed by Mangoum (75.2 ± 7.7%) and Nkolondom (86.1 ± 7.4) (Fig. 3B). Unfortunately, the low sample size did not allow the testing with acetone in Congo DRC. All these results show that *An. gambiae* populations are more likely to develop chlorfenapyr resistance compared to *An. funestus.* Also, the results show that both absolute ethanol and acetone are comparable solvents for chlorfenapyr and *An. gambiae* from Congo DRC, Ghana, Mangoum (CMR) and Nkolondom (CMR) were found as the most resistant populations.

### Time-course exposure to chlorfenapyr 100 µg/ml

**Figure 4:**
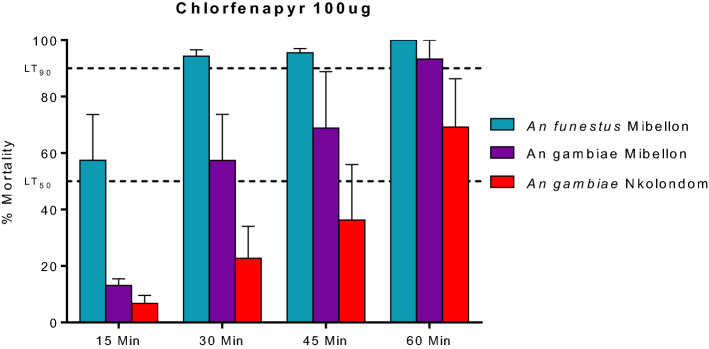
Susceptibility profile of *An. funestus* s.s (Mibellon) and *An. gambiae* (Mibellon and Nkolondom) populations to chlorfenapyr 100 µg/ml at different time points. Error bars represent standard error of the mean. *Min* minutes.

In addition to the previous tests, we performed bioassays with *An. funestus* (Mibellon) and *An. gambiae* (Mibellon and Nkolondom) to chlorfenapyr 100 µg/ml varying the exposure time (15 min, 30 min, 45 min and 60 min). This test revealed that the LT90 is reacheable within the 30 min of exposure for *An. funestus* whereas the LT50 was obtained with 15 min exposure time (Fig. 4). In *An. gambiae* from the same location (Mibellon), the LT90 was obtained only after 60 min exposure and the LT50 after 30 min exposure. In Nkolondom *An gambiae*, the strength of resistance was higher with LT90 not obtained within the 60 min exposure period and the LT50 above 45 min of exposure. All this results confirm a greater risk of chlorfenapyr resistance in *An. gambiae* populations than in *An. funestus.*

### Effect of piperonyl butoxide (PBO), di-ethyl maleate (DEM) and s,s,s-tri-butylphosphorotrithioate (DEF) on susceptibility to chlorfenapyr

**Figure 5:**
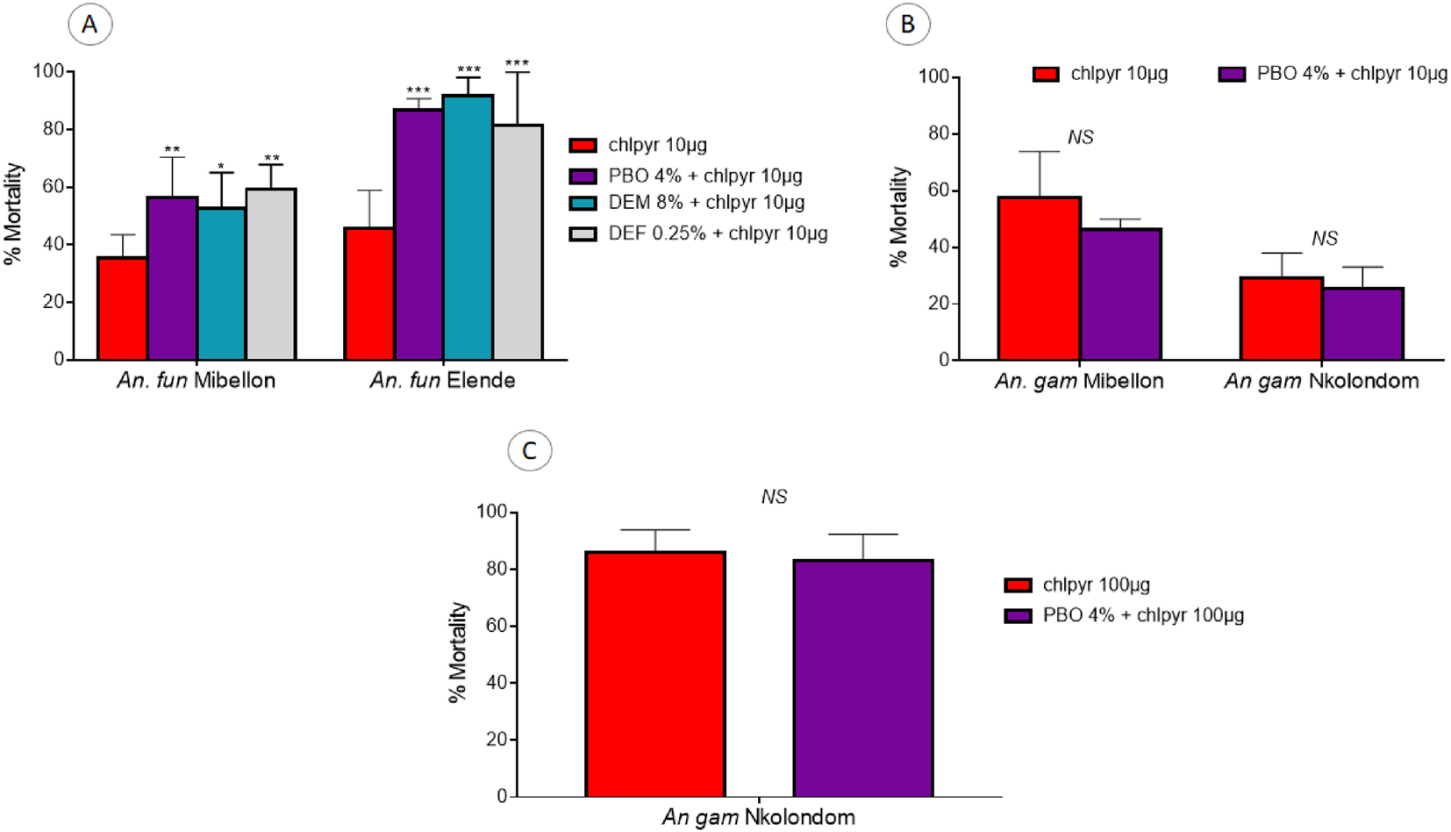
Results of tests with PBO, DEM and DEF. Effect of pre-exposure to inhibitors PBO, DEM and DEF against chlorfenpayr 10 μg/ml for *An. funestus* (from Mibellon and Elende) and *An. gambiae* (from Mibellon and Nkolondom) and 100 μg/ml for *An. gambiae* from Nkolondom. Results are average of percentage mortalities from four-five replicates each. The bars represent the standard error on the mean (SEM), * indicate the level of significance and *NS* represents no significant difference.

With the greater susceptibility observed with all the *An. funestus* populations, we performed bioassays with the inhibitors of P450s (PBO), GSTs (DEM) and ESTs (DEF) to assess the impact of these enzyme systems on the susceptibility to chlorfenapyr. Bioassays conducted on *An. funestus* from Mibellon and Elende revealed a significant recovery of susceptibility to chlorfenapyr 10 µg/ml in both populations tested for all the three inhibitors (Fig. 5). However, the restoration of susceptibility was higher in *An. funestus* from Elende compared to those from Mibellon. In *An. funestus* from Elende, the mortality rate obtained with chlorfenapyr 10 µg/ml (45.8 ± 13.1%) increased significantly after pre-exposure to PBO (χ^2^ = 22.9; P < 0.0001), DEM (χ^2^ = 30.2; P < 0.0001) and DEF (χ^2^ = 16.5; P < 0.0001) as summarised in Fig. 5. In *An. gambiae* populations where resistance was observed, pre-exposure to PBO revealed a slight reduced level of mortality to the same dose of chlorfenpyr (10 µg) but with no significant difference for Mibellon (χ^2^ = 1; P = 0.2) and Nkolondom (χ^2^ = 1; P < 0.5). The same trend was observed with chlorfenapyr100 µg (χ^2^ = 0.2; P = 0.6). This is indicative of a limited effect of P450s on chlorfenapyr potency in *An gambiae.*

### Association between pyrethroid resistant markers and susceptibility to chlorfenapyr

**Figure 6:**
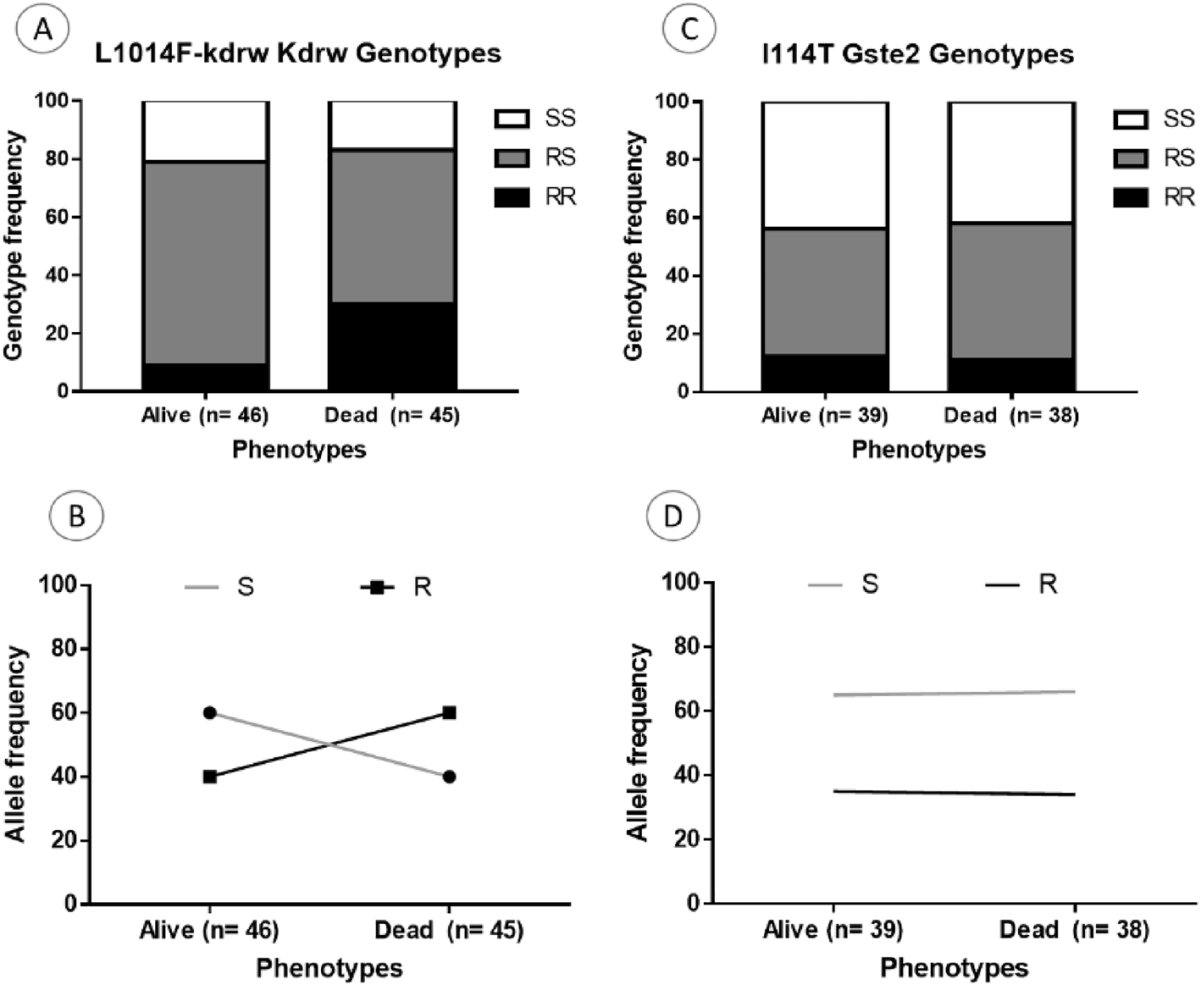
Association between pyrethroids resistance markers and resistance to chlorfenapyr in *An. gambiae*. Distribution of the L1014F-Kdr_w genotypes (**A**) and alleles (**B**) among the dead and alive mosquitoes after exposure to chlorfenapyr and (**C**) and (**D**) for I114T-GSTe2. R represents the resistant allele while S represent the susceptible allele.

Mosquitoes with the 1014F-resistant allele were more susceptible to chorfenapyr exposure indicating a negative cross-resistance between the L1014F-Kdr_w marker and chlorfenapyr resistance. The distribution of L1014F-Kdr_w genotypes in alive hybrid Nkolondom/Kisumu (F4) after exposure with a sub-lethal of chlorfenapyr (10 µg/ml) was as follows: 8.7% (4/46) homozygous resistant (1014F/F), 69.6% (32/46) heterozygotes (L1014F-RS) and 21.7% (10/46) homozygous susceptible (L/L1014) (Fig. 6A,B). In the dead mosquitoes, 28.9% (13/45) were homozygous resistant (1014F/F), 53.3% (24/45) were heterozygotes (L1014F-RS) whereas 17.4% (8/45) were homozygous susceptible (L/L1014). A significant difference was observed in the distribution of L1014F-Kdr_w genotypes between alive and dead mosquitoes (χ^2^ = 13.3; P < 0.001) with high proportion of RR in the dead compared to the alive (χ^2^ = 5.9; P = 0.01). However, no difference was found in the genotype frequency of SS in the alive compared to the dead (χ^2^ = 0.3; P = 0.5) same for the heterozygote (χ^2^ = 2.5; P = 0.1). At the allelic level, the frequency of the 1014F allele was low in the dead compared to alive (χ^2^ = 3.6; P = 0.05). Assessment of the odds ratio revealed that mosquitoes with the RR genotype have less chance to survive compared to RS (OR 0.2; CI 0.09–0.5; P = 0.0004) and SS (OR 0.2; CI 0.09–0.6; P = 0.007). No association was found between the I114T-GSTe2 mutation and chlorfenapyr resistance in *An. gambiae* (Fig. 6C,D)*.* For this marker, no significant difference was observed in the genotype distribution between alive and dead ((χ^2^ = 0.2; P = 0.9). This was confirmed by the absence of correlation between RR and SS (OR 1.1; CI 0.4–2.8; P = 0.5) as presented in Table 1.

**Table 1:** Association between pyrethroids resistance markers and resistance to chlorfenapyr in.

Combination of genotypes at various locus	*An. gambiae*		*An. funestus*
	L1014F-KDR W	I114T-GSTE2	L119F-GSTE2
RR VS RS	0.2 (0.09–0.5) P = 0.0004	1.2 (0.5–3.1) P = 0.4	1.5 (0.8–2.8) P = 0.1
RR VS SS	0.2 (0.09–0.6) P = 0.007	1.1 (0.4–2.8) P = 0.5	2.8 (1.3–7.3) P = 0.02
RS VS SS	1.4 (0.5–2.16) P = 1	0.8 (0.5–1.6) P = 0.4	1.9 (0.8–4.5) P = 0.1
R VS S	0.4 (0.2–0.9) P = 0.003	1.01 (0.5–1.8) P = 0.5	1.5 (0.8–2.6) P = 0.1

**Figure 7:**
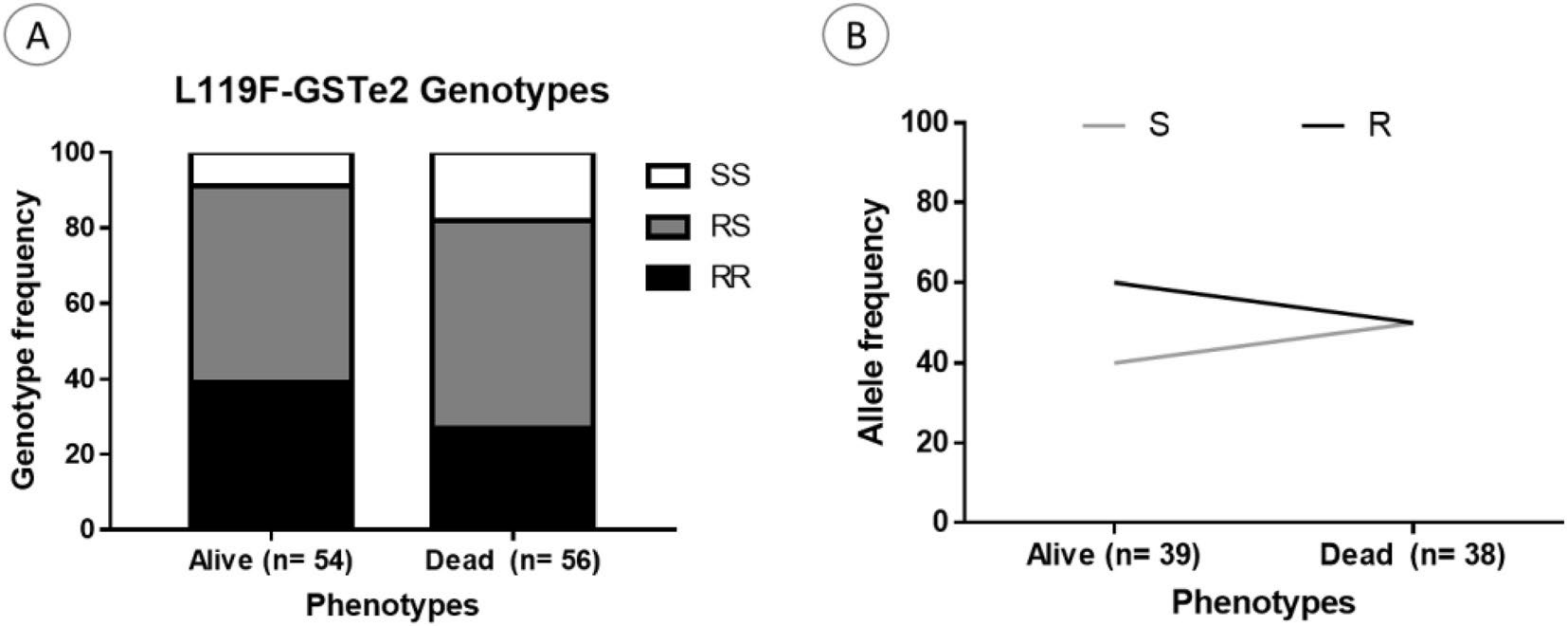
Association between the L119F-GSTe2 mutation and resistance to chlorfenapyr in *An funestus. *Distribution of genotypes and alleles among the dead and alive mosquitoes after exposure to chlorfenapyr. R represents the resistant allele while S represents the susceptible allele.

In *An. funestus* from Elende, analysis of the association between the L119F-GSTe2 mutation and the ability to survive chlorfenapyr exposure revealed that mosquitoes with the homozygote resistant genotype (RR) had a significant ability to survive compared to SS (OR 2.8 CI 1.3–7.3; P = 0.02) (Fig. 7A,B; Table 1).

## Discussion

The present study assessed the susceptibility status of the major malaria vectors *An. gambiae* and *An. funestus* to chlorfenapyr across Africa and explored for the first time potential cross-resistance with known pyrethroid molecular markers.

### Vector populations exhibit a variable resistance profile across Africa

Most of the *An. gambiae* populations tested were resistant to chlorfenapyr (100 µg AI/bottle) while all the *An. funestus* were susceptible to this insecticide. The susceptibility to chlorfenapyr observed in this study particularly in *An. funestus* is reported in many other african countries and support the choice of this insecticide for vector control. Large-scale susceptibility testing of this insecticide using the interim discriminating concentration of 100 µg AI/bottle as done in this study against thousands of *An. gambiae* s.l. in 16 countries demonstrated vector susceptibility, including mosquitoes with multiple resistance mechanisms to pyrethroids^7^. Other studies in Faranah Prefecture of Guinea with Chlorfenapyr 100 µg AI/bottle revealed 100% mortality with wild *An*. *gambiae*, while in the Agréby-Tiassa Region of south-east Côte d’Ivoire, the same concentration induced only 95.5% mortality indicating a reduced suceptibility^26,27^. In this study, when using the concentration of 100 µg AI/bottle, we detected well-established resistance levels in *An. gambiae* populations from Cameroon, DR Congo and Ghana showing that this species could rapidly develop chlorfenapyr resistance compared to *An. funestus*. Such ability to develop chlorfenapyr resistance could be associated to agricultural practices as all the *An. gambiae* tested were collected from agricultural settings. *An. gambiae* larvae tend to breed in small temporal water bodies such as puddles and ditches which can be easily contaminated by agricultural pesticides selecting the resistant mosquitoes. However, there is still no evidence that chlorfenapyr is actively been used in these agricultural sites. One cannot also exclude a possible cross-resistance with other pesticides used by farmers. Overall, more investigations are needed to establish the potential source of selection for chlorfenapyr resistance in such populations of malaria vectors. The significant reduced susceptibility level observed to chlorfenapyr in the three localities would be among the first reports of tolerance to this insecticide in malaria vectors. Such reduced susceptibility would suggest that field populations of malaria vectors, notably from *An. gambiae,* may already harbour some genetic factors to withstand exposure to this insecticide and this calls for the design of suitable resistance management plan to anticipate on such resistance. Full susceptibility observed in *An. funestus* populations indicates that chlorfenapyr-based tools could help controlling this species as reported with IG2 in several countries such as Benin and Burkina Faso and Ivory Coast where this net induced 71% and 80% and 87% mortality in *An. gambiae* and *An. funestus* respectively. This high susceptibility of *An. funestus* could also explain the highest efficacy of Interceptor G2 net in the randomised-control trial in Tanzania a region where this species was predominant (94.5%)^28^.

### PBO displays synergistic effect with chlorfenapyr in *An. funestus* but potential antagonistic effect in *An. gambiae* from Cameroon

Because of the significant resistance noticed in some *An. gambiae* populations and the susceptibility observed with all the *An. funestus* populations, we performed bioassays with the inhibitors of P450s (PBO), GSTs (DEM) and ESTs (DEF) to identify the possible enzyme systems impacting susceptibility to chlorfenapyr as it was reported that metabolic activity increases the bioactivation of this insecticide increasing mosquito mortality^6^. In *An. gambiae*, PBO-prexposure slightly reduced the mortality of *An. gambiae* to chlorfenapyr 10 µg/ml and 100 µg/ml showing potential antagonistic effect of this P450-inhibitor. Suprisingly, tests conducted on *An. funestus* from Mibellon and Elende revealed a significant increase of susceptibility to chlorfenapyr 10 µg/ml in all the two populations tested for all the three inhibitors. This indicates that CYPs may affect detoxification of chlorfenapyr as observed in silkworms for BmCYP4C1^29^, but further studies are needed to investigate the exact role of CYPs in the processing of this insecticide. A trend of antagonism seeing with PBO on *An. gambiae* was reported in potentiation studies conducted with susceptible and resistant strains of *An. stephensi* with 15% PBO^30^. The same antagonistic effect was also reported in insecticide-susceptible *Ae. aegypti* by Paul et al.^31^. Monooxygenases metabolize chlorfenapyr to its active toxic insecticide form^5^, and was demonstrated in *Tetranychus urticae*, where PBO antagonized 2.3-fold toxicity of chlorfenapyr^32^. This antagonistic effect shows that chlorfenapyr can be activated by cytochrome P450 monooxygenases, and uncoupled oxidative phosphorylation in mitochondria and could therefore improve the efficacy of chlorfenapyr in areas of high P450-based resistance. The contrasting pattern between *An. funestus* and *An. gambiae* could indicate a complexity in mechanisms conferring chlorfenapyr resistance.

### Negative association between L1014F-kdr marker and chlorfenapyr resistance

Understanding the cross-resistance pattern conferring resistance to a particular candidate insecticide is critical to formulate strategies for resistance management^33^. The high levels of susceptibility observed in *An. funestus* field populations to chlorfenapyr indicates a potential lack of cross-resistance with pyrethroid insecticides for the tested populations. This was confirmed for the I114T and L119F-GSTe2 (DDT/pyrethroid resistant marker) mutations for which no correlation was seen with the ability to survive chlorfenapyr exposure. This can be justified by the fact that chlorfenapyr do not have a particular target site like other insecticides. Indeed, chlorfenapyr is considered as pro-insecticide that is activated by oxidase enzymes suggesting a potential for negative cross-resistance^30^ as observed for the *kdr* in this study. Mosquitoes bearing the 1014F-resistant allele were more vulnerable to chlorfenapyr as observed previously with clothianidin^34^ showing that this insecticide could help controlling kdr-resistant mosquitoes. However, resistance was only detected in *An. gambiae* populations (where the *kdr* is mostly fixed) showing that mechanisms developed for chlorfenapyr resistance in *An. gambiae* are not linked to the *kdr* and need to be investigated. It is also possible that the correlation between *kdr* and resistance to chlorfenapyr here could be caused by a linkage of *kdr* and metabolic resistance and not necessarily a direct action of *kdr* as the chlorfenapyr does not have a particular target. Tests with synergist of pyrethroids increased the level of suscceptibility to chlorfenapyr showing potentially that some detoxification enzymes belonging to cytochrome P450s, GSTs or esterases could also affect chlorfenapyr efficacy as seeing for BmCYP4C1 in silkworms^29^. However, more studies are needed to establish the level of association between pyrethroid resistance and chlorfenapyr efficacy in major malaria vectors or to characterise the molecular basis of chlorfenapyr tolerance observed in *An. gambiae*. This will allow to predict the potential for cross-resistance and spread of chlorfenapyr resistance and help guiding National Malaria Control Programmes in the selection of insecticides for ITNs and IRS.

## Conclusions

This study showed that although the *kdr-resistant* mosquitoes were more susceptible to chlorfenapyr with limited role of GSTe2-based resistant markers, *An. gambiae* is more likely to develop chlorfenapyr resistance compared to *An. funestus*. This indicates that chlorfenapyr resistance needs to be well monitored and mechanisms driving resistance to this new insecticide class elucidated at the early stage to help prolong the effectiveness of chlorfenapyr-based tools such as interceptor G2.

## Supplementary Information

Supplementary Information.

## Data Availability

All data generated or analysed during this study are included in this published article and its supplementary information files.
