# Supplementary material for: Detection of a reduced susceptibility to chlorfenapyr in the malaria vector Anopheles gambiae contrasts with full susceptibility in Anopheles funestus across Africa
Source: Sci Rep. 2023 Feb 9;13:2363. doi: 10.1038/s41598-023-29605-w (PMC9911381; doi:10.1038/s41598-023-29605-w)
Supplement: Supplementary file 1 — Supplementary Information. [file 41598_2023_29605_MOESM1_ESM.docx]

**Table S1: Comparative susceptibility status of *An. gambiae* and *An. funestus* to Chlorfenapyr 100µg/ml diluted in acetone and absolute ethanol**

| Locality | Species | Mortality rate (%) | | Status |
| --- | --- | --- | --- | --- |
|  |  | **Ethanol 100%** | **Acetone** |  |
| Nkolondom (CMR) | *An. gambiae* | 74.3 ± 12.6  (n=120) | 86.1 ± 7.4 (n=91) | Resistant |
| Mangoum (CMR) | *An. gambiae* | 65.7 ± 18.5  (n=69) | 75.2 ± 7.7 (n=64) | Resistant |
| Elende (CMR) | *An. funestus* | 90.7 ± 7.7  (n=60) | 100 (n=100) | Susceptible |
| Njombe-penja (CMR) | *An. funestus* | 90.7 ± 7.7  (n=49) | 98.8 ± 1.2 (n=70) | Susceptible |
|  | *An. coluzzii* | NT | 100  (n=80) | Susceptible |
| Mibellon (CMR) | *An. gambiae* | 75.6 ± 8.2  (n=64) | 93.3 ± 6.7 (n=74) | Resistant |
|  | *An. funestus* | 100  (n=65) | 100  (n=68) | Susceptible |
| Ndjili (DRC) | *An. gambiae s.l* | 64.3 ± 7.1  (n=42) | NT | Resistant |
| Chikwawa (MWI) | *An. funestus s.l* | 100  (n=84) | 100  (n=100) | Susceptible |
| Atatem (GHA) | *An. gambiae*  *s.l* | 87.9 ± 9.9  (n=48) | 65.9 ± 7.4 (n=63) | Resistant |
|  | *An. funestus* s.s | 100  (n=75) | 100  (n=74) | Susceptible |
| Mayuge (UGA) | *An. gambiae* s.l | NT | 98.3+1.7 (n=60) | Susceptible |
|  | *An. funestus* | NT | 100  (n=80) | Susceptible |
